# Late-Onset Isolated Corticotrope Deficiency in a Woman with Down Syndrome

**DOI:** 10.1155/2021/5562831

**Published:** 2021-04-27

**Authors:** Ibtissem Oueslati, Marwa Ben Jemaa, Meriem Yazidi, Fatma Chaker, Melika Chihaoui

**Affiliations:** Department of Endocrinology, La Rabta University Hospital, University of Tunis-El Manar, Faculty of Medicine, Tunis, Tunisia

## Abstract

Isolated corticotrope deficiency is a rare cause of secondary adrenocortical insufficiency. Its occurrence in patients with Down syndrome is exceptional. Herein, we report a case of an isolated corticotrope deficiency diagnosed at the age of 33 years in a woman with Down syndrome and discuss its possible mechanisms. A 33-year-old woman with Down syndrome was referred to our department for the investigation of low blood pressure. She complained of asthenia, dizziness, and palpitation with arterial hypotension for the past 4 years. The thyroid function was normal and anti-thyroperoxidase antibodies were negative. The peak of cortisol level in response to the insulin-induced hypoglycemia test was 9.4 *μ*g/dl. ACTH level was normal, indicating corticotrope deficiency. Other pituitary hormones were normal. Magnetic resonance imaging scan revealed a partially empty sella turcica. Genetic analysis showed no mutations and no copy number variants of the TBX19 and NFKB2 genes. The mechanism of isolated corticotrope deficiency is unclear, but it may be induced by autoimmune mechanism in similar to other disorders of patients with Down syndrome.

## 1. Introduction

Down syndrome (DS) is the most common genetic abnormality resulting from the trisomy of chromosome 21. It affects one in 500 to one in 1,000 live births. Patients with Down syndrome present with morphological abnormalities, a varying degree of intellectual disability, and other comorbidities such as congenital heart disease, celiac disease, and endocrinopathies. The most common endocrine dysfunctions in patients with Down syndrome are Hashimoto's thyroiditis, Graves' disease, obesity, and diabetes mellitus [1].

Isolated corticotrope deficiency is a rare cause of secondary adrenocortical insufficiency. It is defined as low or absent adrenocorticotropic hormone (ACTH) production with normal secretion of other pituitary hormones and the absence of structural defects within the pituitary gland [2]. To the best of our knowledge, its occurrence in patients with Down syndrome was reported in only one case [3].

Herein, we report a case of an isolated corticotrope deficiency diagnosed at the age of 33 years in a woman with Down syndrome and discuss its possible mechanisms.

## 2. Case Presentation

A 33-year-old woman with Down syndrome was referred to our department for the investigation of low blood pressure. She was born, at term, to nonconsanguineous parents with no history of birth trauma, neonatal hypoglycemia, and prolonged jaundice. Her past medical history was unremarkable apart from surgery for tympanic imperforation. Her age at menarche was 12 years and she had regular periods. Her family history was notable for type 2 diabetes mellitus and arterial hypertension without any history of autoimmune diseases or congenital hypopituitarism.

The patient complained of asthenia, dizziness, and palpitation with arterial hypotension for the past 4 years. Concomitant capillary glucose levels were about 0.82 and 0.85 g/L. Neither bodyweight loss nor polyuria were reported. She was not taking any drugs.

On physical examination, she had a bodyweight of 64 kg, a height of 153 cm corresponding to a body mass index of 27.3 kg/m^2^, a blood pressure of 90/60 mmHg, and a regular pulse of 75 bpm. The skin was pale without hyperpigmentation. The hydration state and thyroid, abdominal, and neurological examinations were normal.

The results of biological investigations are shown in Table 1. The thyroid function was normal and anti-thyroperoxidase antibodies were negative. The peak of the cortisol level in response to the insulin-induced hypoglycemia test was 9.4 *μ*g/dl. The ACTH level was normal indicating corticotrope deficiency. Other pituitary hormones were normal, consisting with the diagnosis of an isolated corticotrope deficiency.

**Figure 1:**
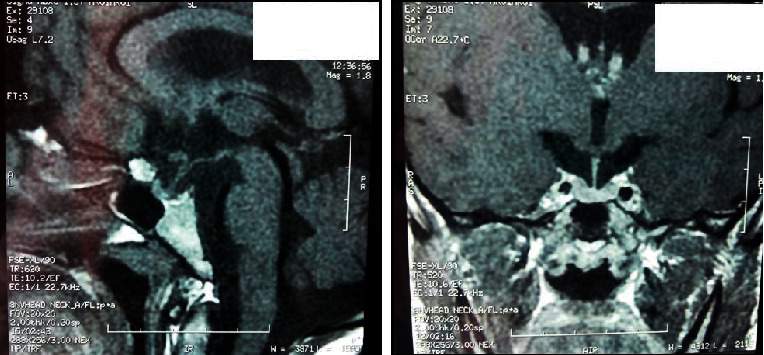
MRI scan showing a small and homogeneous anterior pituitary gland with a partially empty sella turcica.

Magnetic resonance imaging (MRI) scan revealed a small and homogeneous anterior pituitary gland with a partially empty sella turcica. There was no tumor and the posterior lobe bright spot was normal. The pituitary stalk was median and thin (Figure 1).

Genetic analysis using high-throughput sequencing showed no mutations and no copy number variants of the TBX19 and NFKB2 genes. Pituitary antibodies were not available.

Lifelong replacement therapy with an oral hydrocortisone dose of 20 mg per day was prescribed to our patient. The other pituitary hormones remained normal over the course of follow-up.

## 3. Discussion

ACTH that is derived from the cleavage of the precursor polypeptide pro-opiomelanocortin (POMC) is produced by the anterior pituitary gland after stimulation by hypothalamic corticotropin-releasing hormone (CRH). ACTH stimulates the adrenal glands to produce glucocorticoids and adrenal androgens. Corticotrope deficiency results from the decrease or the absence of ACTH production. It is most often associated with other pituitary hormone deficiencies. Isolated corticotrope deficiency is uncommon, especially in adults. Its prevalence is underestimated as it is often misdiagnosed for a long time. In adults, the mean age at disease diagnosis is 50 years and there is a male predominance [4]. In our case, the diagnosis was established at the age of 33 years and the symptoms started at the age of 29 years.

The clinical presentation of isolated corticotrope deficiency is variable and nonspecific. It is insidious in the majority of cases. The acute adrenal crisis is rare. The common chief symptoms are fatigue, weakness, anorexia, and weight loss [5]. Other complaints such as hypoglycemia, hypotension, depression, apathy, and some neurological and musculoskeletal symptoms were also reported [4, 6]. Our patient complained of asthenia, dizziness, and palpitation with arterial hypotension.

Isolated corticotrope deficiency can be caused by several etiologies that can be classified into congenital or acquired forms. In some cases, the etiology remains unknown and the ACTH deficiency is called idiopathic isolated corticotrope deficiency.

The congenital form is more frequent in childhood. Two forms were described. The early-onset form which appears before the age of one or two years and the late-onset form after 3 years [7]. A case of congenital isolated corticotrope deficiency was diagnosed at the age of 29 years [5].

The development of the hypothalamic-pituitary axis depends on the sequential expression of numerous transcription factors and signaling molecules. Genetic mutations in some of these factors can lead to an isolated corticotrope deficiency. The T-box pituitary restricted transcription factor (TBX19) is a member of the T-box gene family that is required for the expression of the POMC gene. Individuals with mutations in TBX19 have a neonatal-onset ACTH deficiency [8]. These mutations were not reported in late-onset forms of isolated corticotrope deficiency.

Corticotrope deficiency can also result from mutations in the POMC gene, and it is in this case associated with early-onset obesity and red hair pigmentation [9]. Moreover, isolated corticotrope deficiency may be associated with variable immune deficiencies in the context of DAVID syndrome (deficit in anterior pituitary function and variable immunodeficiency) due to heterozygous mutations in the *NFKB2* gene [10]. Its pathogenesis in this syndrome remains unclear. In our case, the genetic analysis showed neither mutations nor copy number variants on the *TBX19* and *NFKB2* genes.

The acquired forms of isolated corticotrope deficiency are more frequent in adults. In the absence of steroid-induced adrenal insufficiency, autoimmunity is considered as the primary underlying mechanism for isolated corticotrope deficiency in adults [10, 11]. In our patient, regarding the age of onset, the Down syndrome condition, and the MRI aspect, the most probably incriminated etiology is an autoimmune disorder.

Autoimmune diseases are more frequent in patients with Down syndrome than in the rest of the population. Thyroid autoimmune disorders such as Hashimoto's thyroiditis or Grave's disease are the commonest. These patients are also more predisposed to develop early-onset type 1 diabetes, Addison disease, celiac disease, alopecia areata, chronic autoimmune hepatitis, and primary sclerosing cholangitis [12]. According to many authors, these patients share clinical features, such as autoimmune manifestations and specific autoantibodies, with patients suffering from autoimmune polyendocrine syndrome type 1 [13]. This syndrome results from inactivating mutations in the autoimmune regulator (AIRE) gene, located on chromosome 21. The AIRE acts as a transcriptional regulator that promotes immunological central tolerance by inducing the ectopic thymic expression of many tissue-specific antigens [14]. Its mutations are associated with a huge spectrum of phenotypes. Two major forms were identified. The classical autoimmune polyendocrinopathy candidiasis ectodermal dystrophy (APCED) is an autosomal recessive disease defined by the presence of at least two of the major components including chronic mucocutaneous candidiasis, chronic hypoparathyroidism, and autoimmune Addison's disease [15]. The nonclassical form is caused by dominant heterozygous mutations mainly in the first plant homeodomain (PHD1) zinc finger of AIRE and is characterized by late-onset, milder phenotype, and reduced penetrance [15]. The pituitary involvement in autoimmune polyendocrine syndrome type 1 is a rare condition. Growth hormone deficiency, central diabetes insipidus, gonadotropin deficiency, and ACTH deficiency were reported.

In patients with Down syndrome, Skogberg et al. demonstrated that the AIRE gene is overexpressed, which may alter thymic selection processes and affect the susceptibility for autoimmune diseases [13]. On the contrary, other authors reported that the level of AIRE expression in the thymus of patients with Down syndrome is reduced [16,17].

Corticotrope cells are often the first to be affected in lymphocytic hypophysitis, and adult isolated corticotrope deficiency was suggested to have a link with an autoimmune process [18]. De Bellis et al. demonstrated higher pituitary and hypothalamic antibody titers, respectively, in 14.8 and 18.5% of patients with isolated corticotrope deficiency [19]. Among corticotrope cell antibodies, Tpit antibodies are involved in the development of lymphocytic hypophysitis [20]. Tpit is an essential transcription factor for the development of corticotrope cells. However, this antibody is not specific to lymphocytic hypophysitis, as it was also detected in other autoimmune endocrine diseases [21]. Kiyota et al. identified a novel anterior pituitary autoantigen candidate, Rab GDI alpha, in patients with isolated corticotrope deficiency [22].

To the best of our knowledge, the coexistence of Down syndrome and isolated corticotrope deficiency was reported in only one case [3]. It was an 8-year-old boy with Down syndrome, congenital primary hypothyroidism, and central precocious puberty who presented with fatigue. The basal cortisol level was 7.6 *μ*g/dl, the stimulated cortisol level was 10.2 *μ*g/dl, and the ACTH level was 41.6 pg/ml. The pituitary MRI revealed pituitary gland hypoplasia. The etiology of the isolated corticotrope deficiency was not determined in this case.

## 4. Conclusion

In the absence of steroid-induced adrenal insufficiency, isolated corticotrope deficiency is a rare condition. Genetic forms are most often diagnosed in childhood, whereas in adults it is mainly secondary to an autoimmune disorder. The occurrence of an isolated corticotrope deficiency in patients with Down syndrome is exceptional. Its mechanism is still unclear. It may be induced by an autoimmune disorder.

## Figures and Tables

**Table 1:** Blood biological and hormonal parameters.

	Values	Reference ranges
Fasting glucose (mmol/L)	4.95	3.84–5.44
Creatinine (*μ*mol/L)	61.8	61–106
Sodium (mmol/L)	137	136–145
Potassium (mmol/L)	4	3,5–5,1
Calcium (mg/L)	87	85–105
Phosphorus (mg/L)	29	23–47
White blood cells(mm^3^)	7060	4000–10,000
Lymphocytes	1360	1500–4000
Neutrophils	5210	1500–7000
Hemoglobin (g/dL)	13.1	12–16
TSH (mIU/L)	1.97	0.35–4.95
FT4 (ng/dL)	0.91	0.7–1.5
8h cortisol (*μ*g/dL)	8.4	4–20
Pic cortisol (*μ*g/dL)	9.4	≥18
ACTH (pg/ml)	21.4	10–48
Prolactin (ng/ml)	20	<25
FSH (IU/L)	1.7	3.35–12.6
LH (IU/L)	6.53	2.39–6.6
IGF1 (ng/ml)	290	109–358

## Data Availability

No data were used to support this study.
